# Vaccine Safety: Myths and Misinformation

**DOI:** 10.3389/fmicb.2020.00372

**Published:** 2020-03-17

**Authors:** Sarah Geoghegan, Kevin P. O’Callaghan, Paul A. Offit

**Affiliations:** ^1^Division of Infectious Diseases, The Children’s Hospital of Philadelphia, Philadelphia, PA, United States; ^2^National Children’s Research Centre, Dublin, Ireland

**Keywords:** vaccine, safety, adjuvant, immunization, side effect

## Abstract

The World Health Organization has named vaccine hesitancy as one of the top ten threats to global health in 2019. The reasons why people choose not to vaccinate are complex, but lack of confidence in vaccine safety, driven by concerns about adverse events, has been identified as one of the key factors. Healthcare workers, especially those in primary care, remain key influencers on vaccine decisions. It is important, therefore, that they be supported by having easy access to trusted, evidence-based information on vaccines. Although parents and patients have a number of concerns about vaccine safety, among the most common are fears that adjuvants like aluminum, preservatives like mercury, inactivating agents like formaldehyde, manufacturing residuals like human or animal DNA fragments, and simply the sheer number of vaccines might be overwhelming, weakening or perturbing the immune system. As a consequence, some fear that vaccines are causing autism, diabetes, developmental delays, hyperactivity, and attention-deficit disorders, amongst others. In this review we will address several of these topics and highlight the robust body of scientific evidence that refutes common concerns about vaccine safety.

## Introduction

Vaccines are among the greatest public health achievements of modern times. They have saved and continue to save millions of lives throughout the world. Their success is such that in high income settings most patients, parents and health care providers do not have first-hand experience of the devastating consequences of many of the diseases that they prevent. Over the past few decades vaccine hesitancy has emerged as a major public health problem leading to outbreaks of communicable infections such as measles. The reasons for vaccine refusal are complex and differ according to geographical and cultural context. However, concern about vaccine safety continues to be an important driver of decreased vaccine uptake in most contexts (Larson et al., 2014). This concern is fueled by misinformation and propagated through organized antivaccine groups, social media and celebrity endorsements. Despite a wealth of scientific data supporting the safety of currently recommended vaccines, counteracting false information to convince vaccine hesitant populations continues to be challenging. While there will always be individuals with strongly held beliefs for whom well informed discussions about vaccine safety will have little impact, it has been repeatedly demonstrated that provider recommendation strongly influences decision making around vaccines (Smith et al., 2017). Thus it is important that front line health care workers understand the available safety data and feel empowered to give strong advice backed by scientific evidence. In this article we aim to discuss the most common vaccine safety controversies, summarize the available data, and highlight some of what we feel are the most important research studies for each topic (Table 1).

**TABLE 1:** Key concepts addressing common vaccine safety concerns.

**Common vaccine safety myths**	**Key concepts**
Too many vaccines too soon.	• The number of immunologic components in vaccines have declined over time. • The current 14 vaccines on the United States schedule contain 200 immunologic proteins in total, the smallpox vaccine contained 160.
Too many vaccines can “overwhelm” the immune system.	• Epidemiologic data and biologic data show that cumulative increases in the number of vaccines have no effect on immune function.
MMR vaccine causes autism.	• Original study making this claim contained 12 children, the paper was subsequently retracted due to evidence of misrepresented data. • Multiple large scale studies, including a study of half a million children have shown no association between receipt of MMR and risk of autism.
HPV vaccine increases risk of autoimmune disease.	• More than 270 million doses of HPV vaccine have been administered. • Repeated well-designed studies show no association between HPV and AI disease.
Influenza vaccine given in early pregnancy increases risk of miscarriage.	• A study of 2762 women showed no association between influenza vaccine and spontaneous abortion.

## Do Vaccines Cause Autism?

The speculated link between measles, mumps, rubella vaccine (MMR) and autism spectrum disorders has a long and complicated history, beginning with a (since retracted) report in the *Lancet* in 1998 (Wakefield et al., 1998). The retraction occurred due to misrepresentation of clinical and biological data in the paper, issues which, in part, led to the lead author, Andrew Wakefield, being removed from the United Kingdom’s medical register. This report described 12 children with a history of pervasive developmental disorder (PDD), 8 of whom had autism, along with intestinal symptomatology. A hypothesis was postulated in this paper that receipt of MMR vaccine had led to intestinal inflammation, with entrance to the bloodstream of proteins that led to brain inflammation and the development of PDD, including autism disorder. The paper was problematic in a number of ways, including that a true comparison between MMR-exposed and unexposed children was not carried out. Additionally, the only correlation between MMR-exposure and autism was report of receipt of MMR during a time period proximate to the diagnosis of autism. Since, in the natural history of the disease, the development of symptoms of autism can be expected to occur at a similar age to receipt of MMR, this apparent correlation is not surprising. Lastly, although the postulated sequence of pathogenesis had intestinal inflammation as an intermediate step, all 8 cases of autism had intestinal symptoms that started after the diagnosis of autism, not before.

Subsequent to the publication of this report, and although it was later retracted, the article led to broad media coverage of the postulated link, and widespread concern as to the safety of MMR vaccination developed. In this setting, MMR vaccine coverage in the United Kingdom and elsewhere dropped precipitously, leading to the re-emergence of measles as a public health problem and associated morbidity and mortality.

Large epidemiological studies, in particular the two referenced herein, have since clearly refuted the postulated link, and have substantively affirmed that vaccination, and the MMR vaccine in particular, have neither a correlative nor causative link to autism spectrum disorders. In 1999, 1 year after the *Lancet* paper, a separate retrospective cohort study of about 500 children was carried out in the North Thames region of England, examining autism spectrum disorders in children both before and after the introduction of the MMR vaccine in 1988 (Taylor et al., 1999). This study detected no differences in the rate of immunization of those children with a diagnosis of autism, as compared with those without this diagnosis. Similarly, no difference was observed in the age of diagnosis of autism between vaccinated and unvaccinated children.

A larger retrospective cohort study, and to date one of the most definitive findings of no causal link between MMR vaccine and autism, was performed by a Danish group and published in the *New England Journal of Medicine* in 2002 (Madsen et al., 2002). The authors reviewed the records of more than half a million children born in Denmark during the study period (1991–1998) and found no association between age at vaccination, receipt of vaccination, or time passed since vaccination, and the development of autism spectrum disorders.

## Are Too Many Vaccines on the Schedule?

The first vaccine was introduced by Edward Jenner in 1796 and ultimately led to the worldwide eradication of smallpox (Riedel, 2005). Currently licensed vaccines are available that prevent 26 different human pathogens. Depending on geographic region children can be recommended to receive more than 30 doses of vaccines protecting as many as 20 different diseases in the first 24 months of life (World Health Organization [WHO], 2019). Children receive the most vaccines during the first year of life at a time when they are most vulnerable to the devastating consequences of invasive bacterial infection such as pneumococcal or *Haemophilus influenza*e meningitis. The increasing number of vaccines on the schedule has led to concerns among some vaccine hesitant groups about effects on a child’s immune system and neurodevelopment. This has in turn led to the dangerous practice of individualized schedules. Timing of the recommended vaccine schedule takes into account the timing of waning of maternal antibody and maturation of the immune system, susceptibility to the disease, and effectiveness and dosing of the vaccine. Vaccines on the schedule have been tested in their final formulations and with other vaccines given at the same time. By addressing concerns with a non-scientific approach such as delaying or separating vaccines, providers with alternative schedules are legitimizing unfounded safety concerns and putting their patients at unnecessary risk (Offit et al., 2002; Offit and Moser, 2009).

While the number of vaccines has increased significantly over time, the numbers of immunologic components in vaccines have declined. Whereas the smallpox vaccine contained about 200 proteins, the 14 recommended vaccines on the United States schedule, combined contain about 160 immunologic components (i.e., viral proteins, bacterial proteins, and bacterial polysaccharides) (Offit et al., 2002). Three factors account for this decline: first, the worldwide eradication of smallpox obviated the need for that vaccine; second, advances in protein chemistry and protein purification; and third, the birth of recombinant DNA technology have resulted in vaccines containing fewer antigens.

## Can Receiving Multiple Vaccines Weaken the Immune System?

Epidemiologic and immunologic data refute the concept that vaccines dampen the immune response. In a large Danish cohort study comprising 805,206 children born between 1990 and 2001 no adverse associations were observed between an increasing number of vaccinations and hospitalizations with non-vaccine-targeted infections (Hviid et al., 2005). A second nested case control study included 944 patients from 6 United States healthcare organizations to examine cumulative vaccine antigen exposure in the first 24 months of life and risk of non-vaccine-targeted infections from 24–47 months. The group found no differences in cumulative vaccine antigen exposure between children who were admitted to hospital with a non-vaccine targeted infection and those who were not (Glanz et al., 2018). The epidemiologic data is supported by biological evidence. A study in Canada investigating the impact of vaccination on immune status compared the immune response to general non-antigen-specific stimuli in entirely unvaccinated versus vaccinated children at 3–5 years of age. Innate and adaptive responses were compared. Investigators did not find any differences in immunological outcomes in vaccinated children. Furthermore, and notably, equivalently robust innate and adaptive responses to pathogen associated microbial patterns and generic T-cell stimulants were seen in both groups (Sherrid et al., 2017).

## Does Giving Vaccines in the First 24 Months of Life Impact Neurodevelopmental Outcome?

Facing the large body of safety data demonstrating no link between MMR vaccine and autism, a new concern frequently cited by antivaccine groups is that the number of antigens children are exposed to in the first year of life has an adverse effect on neurodevelopment. This has also been well studied and there has been no correlation found between the number of vaccine antigens received and adverse neuropsychological outcomes (Iqbal et al., 2013), nor has a difference been demonstrated in neuropsychological outcomes in those who received vaccines on time in the first year of life compared to delayed schedules (Smith and Woods, 2010).

## Are the Adjuvants and Preservatives Used in Vaccines Safe?

Since large-scale epidemiological studies have broadly disproven any link between the vaccine antigens and development of adverse neurodevelopmental outcomes, more recent vaccine hesitancy efforts have centered around concerns as to the safety of a variety of vaccine ingredients – including adjuvants and preservatives. Two of the more commonly cited of these are aluminum and mercury.

Aluminum is the third most abundant element in the environment, and the most abundant metal, and is found diffusely in soil, water, plants, and air. It is found in a variety of consumer products, as well as throughout the human food chain, and in many pharmaceuticals. In vaccines, aluminum is used as an adjuvant, a component that boosts immune response to the vaccine antigens. When adjuvants are used, they allow for smaller amounts of vaccine to be given, as well as fewer doses. Aluminum has been used in a variety of vaccines, including hepatitis A and B, *H. influenzae* type b, and pneumococcal vaccines.

As a vaccine component, aluminum has been extensively tested for safety as part of pre-licensure clinical trials. We know from studies examining the aluminum exposure of infants that the cumulative amount of aluminum from vaccines in the first 6 months of life is actually far less than that received from dietary sources, including both breast milk and formula (Keith et al., 2002; Mitkus et al., 2011). Both sources represent far less exposure than that represented by a regulatory minimal risk level (MRL), which is established by the Agency for Toxic Substances and Disease Registry. The safety of aluminum salts from vaccines, and from other sources, has also been established in studies examining blood and hair levels of aluminum in infants, and associated neurodevelopmental outcomes (Karwowski et al., 2018). Such studies have not proven a link between blood and hair concentrations of aluminum and receipt of vaccines, nor between blood and hair aluminum concentrations and neurodevelopmental outcomes.

Mercury, or more specifically a compound containing ethylmercury called thimerosal, has been used at various points in the past as a preservative in multidose vials of vaccine. Because some forms of mercury are known to be neurotoxic, this has led to concerns in the past that this mercury-containing compound could represent a danger to patient safety. However, the form of mercury contained in thimerosal (ethylmercury) is substantially different from the more toxic methylmercury, despite the similarity in their names. Ethylmercury is cleared quickly from human tissues, and does not accumulate substantially, unlike methylmercury. When the safety of thimerosal as a vaccine component has been examined, it has not been found to have any associated risks, including no evidence of an increased risk of autism (Tozzi et al., 2009; Price et al., 2010).

## Do Vaccines Contain Human and Animal DNA Fragments?

Due to the fact that some vaccines are manufactured using human embryo cell lines, some residual human DNA can be found in a variety of vaccines, including varicella, rubella, hepatitis A, and one of the rabies vaccines. The exposure of vaccinated patients to such DNA has been raised as a potential safety concern. There are a number of reasons to believe that this exposure does not present a danger to vaccine recipients.

Primary amongst these reasons is the fact that human DNA is highly sensitive to destruction by chemical processes, and much of the DNA involved in creating these vaccines is destroyed in the process. The end result contains only minimal amounts of residual DNA, all of it fragmented, and none of it representing a viable genome. Additionally, isolated portions of DNA cannot incorporate themselves into a new genome without many additional processes being involved. In fact, this involves many of the major issues that make gene therapy difficult.

The safety of tiny amounts of residual human DNA has been assessed by at least two investigator groups, who have used both probabilistic modeling and animal models. These studies all generally concur that dose equivalents on the order of millions to trillions of the amount of DNA contained in vaccines would be required before risk of, for example, an oncogenic event, would become appreciable (Wierenga et al., 1995; Yang, 2013).

## Do Vaccines Cause Autoimmune Diseases?

Associations between vaccines and autoimmunity are another frequently cited safety concern and have been heavily studied in relation to a number of different autoimmune (AI) disorders including diabetes mellitus type 1 (DM type 1), Guillain Barré Syndrome (GBS), multiple sclerosis (MS) and other demyelinating disorders. Existing epidemiologic studies have found no associations between the number of different vaccines and an increased risk of autoimmune disorders. Two recent systematic reviews looking at the association between different individual vaccines and central demyelinating disorders (hepatitis B, human papilloma virus (HPV), influenza, MMR, varicella, tetanus, Bacillus Calmette Guérin (BCG), polio or diphtheria) concluded that there was no relationship between receipt of a vaccine and development of MS (Mailand and Frederiksen, 2017; Mouchet et al., 2018). Links with DM type 1 have also been extensively studied. A German study which included more than 1900 children from a prospective cohort data set found no association between vaccination and development of DM type 1 in children with a high familial risk of AI disorders (Beyerlein et al., 2017). A metanalysis of 23 different case control studies including 11 different vaccines and 13,000 patients found no association between vaccination and risk of DM type 1 (Morgan et al., 2016).

The Advisory Committee on Immunization Practices (ACIP) give a precautionary recommendation on influenza vaccine for patients with a history of Guillain Barré Syndrome within 6 weeks of receiving the vaccine due to data suggesting a possible marginal increased risk. The data on this association is variable and has been detected in some seasons but not in others. A metanalysis of 39 controlled observational studies found that the relative risk of GBS following receipt of any influenza vaccine was 1.41 (Martin Arias et al., 2015). The Vaccine Safety Datalink team, in a self-controlled risk interval analysis of data from the 2009–2010 influenza season found an association between risk of GBS and the monovalent inactivated vaccine (MIV) against H1N1 but not in association with the trivalent inactivated vaccine (TIV) (Greene et al., 2012). However, in a later analysis that included the 2010–2011 season and adjusted for antecedent respiratory infection there was no increased risk following 1.27 million 2009-10 MIV or 2.8 million 2010-11 TIV doses (Greene et al., 2013). The risk of GBS within 6 weeks of influenza infection is greater than after the vaccine. A Canadian study which used a self-controlled risk interval design looked at 2831 admissions for Guillain-Barré syndrome; 330 received an influenza vaccine and 109 had an influenza-coded health-care encounter within 42 weeks before hospitalization. The attributable risks of admissions for GBS within 6 weeks were 1⋅03 per million vaccinations, compared with 17⋅2 GBS admissions per million influenza-coded health-care encounters (Kwong et al., 2013).

In recent years, the HPV vaccine has been a particular subject of concern. The misinformation surrounding HPV vaccines has tragically resulted in lower uptake in many countries, denying millions of adolescents the opportunity to be protected from fatal HPV-related cancers in later life. Meanwhile in Australia, where high uptake has been maintained, a recent modeling study indicates progress toward eliminating cervical cancer as a public health problem (Hall et al., 2019). Since entering the market in 2006 more than 270 million doses of the HPV vaccine have been administered worldwide, including 100 million in the United States (Centers for Disease Control and Prevention [CDC], 2019). Studies have repeatedly demonstrated that this is a safe vaccine and there are strong epidemiologic data refuting claims of an association with AI diseases. These include (most prominently) a large population-based cohort in Denmark and Sweden that analyzed more than 696,000 doses of HPV-4 vaccine and found no evidence of an association with AI disorders (Arnheim-Dahlstrom et al., 2013). Additionally, a large case control study in France matched 211 definite cases of AI disease with 875 controlls. No association was found between HPV vaccine an several AI disorders (i.e., immune thrombocytopenic purpura [ITP], GBS, connective tissue disorders, DM type 1, and autoimmune thyroiditis) (Grimaldi-Bensouda et al., 2017). This study is consistent with the findings of a registry based cohort study in Finland which included 134,615 vaccinated females and (Skufca et al., 2018) a review of 6 years of post-licensure data in the Vaccine Adverse Event Reporting System (VAERS) which identified no unexpected safety concerns (Arana et al., 2018).

## Are the Vaccines Currently Given in Pregnancy Safe?

While vaccines have significantly contributed to a reduction in child mortality, progress in reducing deaths in infants that are too young to receive vaccines has been slower. Administration of vaccines to expectant mothers has the potential to protect the most vulnerable infants from serious illness and death as well as protecting women themselves from increased morbidity and mortality during a time of altered immune function. Influenza and pertussis vaccines (Tdap) are recommended to be given during pregnancy in most high-income countries. Tetanus vaccine remains a recommendation in settings where there is an ongoing risk of neonatal tetanus. Recent data from the CDC indicate that from 2010–2018 pregnant women accounted for 24–34% of influenza-associated hospitalizations per season among women aged 15–44 years and a total of 3,928 pertussis-related hospitalizations among infants aged <2 months (Lindley et al., 2019). Despite this clear burden of disease vaccination rates during pregnancy remain poor. Influenza and Tdap vaccination coverage rates during pregnancy in the United States reported in April 2019 were 53.7 and 54.9%, respectively (Lindley et al., 2019). Safety concerns and provider recommendation are among the most important factors influencing vaccine uptake in pregnancy (Wilson et al., 2015).

Influenza and pertussis vaccines during pregnancy are recommended by both federal agencies and non-governmental advisory groups. Because millions of pregnant women have received the influenza vaccine during pregnancy there is a large body of data confirming that it is safe. Numerous well-designed cohort studies have found no association between influenza vaccine and adverse pregnancy and infant outcomes including birth malformations, still birth, spontaneous abortion, low birth weight and low Apgar scores (Kharbanda et al., 2013, 2017; Zerbo et al., 2017). Impact of timing of vaccination including risk of administration in the first trimester has also been studied and repeatedly shown to be safe (Kharbanda et al., 2017). A critical review of a 2017 study that claimed a theoretical association between influenza vaccine and spontaneous abortion identified a number of methodological flaws including small sample size and in appropriate matching of cases and controls. A follow up study by the same group looking at a larger sample size of 2762 women (three times the amount of the original paper) showed no significant association between spontaneous abortion and influenza vaccine regardless of prior season vaccination status (Donahue et al., 2019). Similarly pertussis vaccine has been shown to be safe in pregnancy in numerous well designed cohort studies (Sukumaran et al., 2015; DeSilva et al., 2016, 2017; Layton et al., 2017). The greatest challenge facing vaccination uptake in pregnancy is not a lack of available safety data but a failure to communicate this adequately to patients. There are currently a number of vaccines in development that are intended to be given to pregnant women including vaccines protecting against respiratory syncytial virus, group B *streptococcus*, Zika virus and CMV. Given this landscape it is crucial that there is a focused effort to improve confidence in vaccine safety during pregnancy among both pregnant women and providers (Heath et al., 2017).

## Do Vaccines Have Any Proven Severe or Life-Threatening Side Effects?

Vaccines are given to healthy people and thus are held to an even higher safety standard than medicines used to treat diseases. They undergo rigorous safety and efficacy studies in the pre-licensure stage. In order to identify rare side effects that may have been missed in pre-licensure studies vaccines undergo ongoing post-licensure monitoring. The infrastructure in place provides robust data about rare side effects of vaccines. Anaphylaxis is example of an established rare but life threatening side effect of vaccines with the most recent United States data from the vaccine safety data link identifying a rate of anaphylaxis of 1.31 (95% CI, 0.90–1.84) per million vaccine doses and no deaths (McNeil et al., 2016).

When considering very rare side effects of vaccines a balance should be sought between the risk of a side effect occurring and the risk of disease occurring if the vaccine is not given. An illustrative example is the use of the oral polio vaccine (OPV). Vaccine strain-related paralytic polio will occur in about 1 in 2.5 million people who receive this vaccine. The oral polio vaccine was used in the United States for 40 years, leading to eradication of the disease in 1979. Since the year 2000 the inactivated polio vaccine (IPV) has been the only polio vaccine recommended for use in the United States (Centers for Disease Control and Prevention [CDC], 2019). This decision was made because the potential, however, rare, for paralysis due to OPV, was considered unacceptable when a feasible alternative existed. However, in many low-income settings, IPV was not a feasible alternative and thus the risk equation was different. OPV is cheaper and more convenient to administer and has led to the near global eradication of disease. The number of cases of wild type polio worldwide has decreased by 99% from 1988 to 2018, from an estimated 350 000 + cases (1988) to 33 reported cases (2018) (World Health Organization [WHO], 2019). Complete eradication worldwide will ultimately require the cessation of use of trivalent OPV, a strategy currently being implemented by the WHO through a synchronized switch to a bivalent oral polio vaccine, successfully implemented in 150 countries as of 2016 (Hampton et al., 2016). Currently, poliovirus types 2 and 3 have been eliminated from the world.

## Conclusion

In this article we summarize common vaccine safety controversies and highlight what we feel are some of the most important studies refuting these concerns. The misinformation surrounding vaccine safety poses a threat to children’s lives worldwide and it is crucial that primary care workers at the front line understand the evidence and can confidently communicate the message that vaccines are a safe and lifesaving intervention.

## Author Contributions

SG and KO’C reviewed the literature and wrote the manuscript. PO designed, reviewed, and edited the manuscript.

## Conflict of Interest

The authors declare that the research was conducted in the absence of any commercial or financial relationships that could be construed as a potential conflict of interest.
